# Bunyaviruses in human, animal and mosquito samples from southeast Austria

**DOI:** 10.1186/1756-3305-7-S1-P14

**Published:** 2014-04-01

**Authors:** HP Huemer, B Seidel, P Hufnagl, A Deutz, A Posautz, S Dowall, R Hewson, Z Hubalek, F Allerberger

**Affiliations:** 1 Dpt. Hygiene, Microbiology & Social Medicine, Medical University Innsbruck, Austria; 2 Ecology and Landscape Assessment, Persenbeug, Austria; 3 Austrian Agency for Health and Food Safety (AGES), Vienna, Austria; 4 Styrian Public Health Authority, BH Murau, Austria; 5 Inst.Wildlife Ecology, University for Veterinary Medicine, Vienna, Austria; 6 Public Health England, Microbiol.Services, Porton Down, UK; 7 Inst. Vertebrate Biology, Academy of Sciences, Brno, Czech Republic

## 

In Austria occurrence of 40 different species of mosquitoes belonging to 6 genera has been described. AGES in 2011 initiated a nation-wide mosquitoe-surveillance program to identify invading species. As part of this project, pools of Culex, Aedes, Anopheles and Culisetta species collected in different parts of the country were analyzed by PCR analysis; in addition seroepidemiological testing for bunyaviruses was performed in selected regions. In order to be able to detect also new strains, a broad spectrum CODEHOP (Consensus-Degenerate Hybrid Oligonucleotide Primer) approach was used initially; presumably due to the low sensitivity of degenerated primer designs and due to dilution in insect pools, we did not pick up any new isolates so far using Orthobunyavirus, Phlebovirus or Nairovirus group-specific primers. Two bunyaviruses were found in Culex pipiens from the southern province Carynthia in 2012, detected with non-degenerate multiplex PCR, the sequences highly homologous to Italian Tahynavirus (TAHV) isolates. 245 sera collected for a study of zoonotic infections in hunters, veterinarians, farmers, and abattoir workers (conducted by the Styrian health authorities in 2003), were tested by ELISA tests, using inactivated Crimean Congo virus (CCHF) from Bulgaria as well as recombinant CCHF nucleoprotein produced in baculovirus. In addition, cell culture derived TAHV lysates as well as TAHV infected cells were used as antigens in ELISA and immunofluorescence (IF) under an experimental setup. No seropositivity for CCHF or related nairoviruses was found in these Austrian human sera originating from risk groups for zoonotic infection.

